# The Incremental Prognostic Value of Hyperemic Coronary Flow Velocity in Patients with Angina and Nonobstructive Coronary Artery Disease

**DOI:** 10.1002/mco2.70731

**Published:** 2026-04-12

**Authors:** Quande Liu, Guihua Jiang, Mingjun Xu, Jichen Pan, Chenghu Guo, Yichun Zhou, Meng Zhang, Yu Zhang, Yun Zhang, Mengmeng Li, Mei Zhang

**Affiliations:** ^1^ National Key Laboratory for Innovation and Transformation of Luobing Theory Jinan China; ^2^ The Key Laboratory of Cardiovascular Remodeling and Function Research Chinese Ministry of Education Chinese National Health Commission and Chinese Academy of Medical Sciences Jinan China; ^3^ Department of Cardiology Qilu Hospital of Shandong University Jinan China

**Keywords:** angina with nonobstructive coronary arteries, coronary flow velocity reserve, coronary microvascular dysfunction, hyperemic coronary flow velocity, transthoracic Doppler echocardiography

## Abstract

Risk stratification in patients with angina and nonobstructive coronary arteries (ANOCA) remains suboptimal. Coronary flow velocity reserve (CFVR) is prognostic but susceptible to hemodynamic variability; we evaluated whether hyperemic coronary flow velocity (hCFV) improves risk prediction. We analyzed 246 consecutively enrolled ANOCA patients and an independent validation cohort (*n* = 135). Transthoracic Doppler of the mid‐distal LAD quantified CFVR and hCFV. The primary end point was major adverse cardiovascular events (MACE). During a median follow‐up of 28.8 months, 27 patients (10.9%) experienced MACE. Both CFVR and hCFV were significantly associated with MACE. Among patients with CFVR < 2.5, hCFV ≤ 0.44 m/s independently predicted MACE (adjusted HR 6.6, *p* = 0.001). A combined CFVR‐hCFV scheme yielded graded risk of MACE (Group A: CFVR ≥ 2.5; Group B: CFVR < 2.5 with hCFV > 0.44 m/s; Group C: CFVR < 2.5 with hCFV ≤ 0.44 m/s), with Group C exhibiting the highest risk of MACE (35.5% vs. 6.3%, 10.5%, *p* < 0.01). Adding reduced hCFV to a model including clinical risk factors and CFVR improved prediction (IDI 0.05, *p* = 0.011; NRI 0.23, *p* = 0.0023) and was confirmed in the validation cohort. Reduced hCFV provides incremental prognostic value beyond CFVR and offers a practical approach to identify high‐risk ANOCA patients.

## Graphical abstract

Patients were first split by CFVR (≥ 2.5 vs. < 2.5). Among those with CFVR < 2.5, peak hyperemic CFV (hCFV) further defined Group B (> 0.44 m/s) and Group C (≤ 0.44 m/s). This hCFV‐guided split identified the ceiling of risk: Group C showed the highest MACE incidence, while Group B had substantially lower risk, consistent with pseudo‐low CFVR driven by high resting flow.

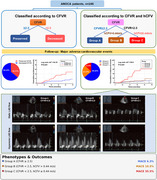

## 1 Introduction

Nearly half of the patients undergoing coronary angiography for angina do not exhibit significant coronary stenosis, a clinical scenario termed angina with nonobstructive coronary arteries (ANOCA) [1]. ANOCA is increasingly recognized as a clinically relevant condition associated with persistent angina, impaired quality of life, and adverse cardiovascular outcomes [2]. Previous studies have shown that ANOCA is a heterogeneous syndrome attributable to various coronary functional abnormalities, and coronary microvascular dysfunction (CMVD) is one of the major underlying mechanisms. The pathophysiological basis of CMVD lies in structural or functional abnormalities of the coronary microcirculation, leading to impaired regulation of coronary perfusion and an imbalance between myocardial oxygen supply and demand, which may in turn result in ischemia‐like myocardial alterations and related clinical symptoms [3].

Transthoracic Doppler echocardiography enables noninvasive assessment of coronary flow velocity reserve (CFVR), defined as the ratio of hyperemic to resting coronary flow velocity. A reduced CFVR (typically defined as CFVR < 2.5 in Doppler echocardiography) has been established as an important noninvasive indicator of coronary microvascular dysfunction (CMVD), as it reflects impaired coronary microvascular vasodilatory function and associated with adverse clinical outcomes [1].

However, the utility of CFVR is constrained by several factors. Its value can be influenced by baseline hemodynamic conditions, such as heart rate and blood pressure, and it is sensitive to exogenous factors like caffeine and vasoactive medications [4]. Furthermore, patients with low CFVR often exhibit significant heterogeneity, suggesting that CFVR alone may not fully capture the underlying pathophysiological status. While prior invasive studies have proposed different CMVD endotypes (e.g., structural vs. functional), the stability and prognostic value of the invasive metrics used to define them have been questioned [5, 6], highlighting the need for more refined noninvasive risk markers.

To address these limitations, coronary flow capacity (CFC), derived from positron emission tomography (PET), has been proposed as a more comprehensive physiological metric. By integrating hyperemic myocardial blood flow with coronary flow reserve, CFC may better characterize coronary physiological impairment and improve risk stratification compared with coronary flow reserve alone [7, 8]. However, the widespread use of PET‐derived CFC is limited by high cost, radiation exposure, limited availability, and technical complexity, restricting its scalability for routine clinical practice.

Transthoracic echocardiography (TTE) offers an accessible, radiation‐free alternative capable of simultaneously measuring CFVR and hyperemic coronary flow velocity (hCFV), with reasonable concordance to invasive indices [9]. However, CFVR captures vasodilatory reserve as a ratio, hCFV reflects the absolute capacity to augment flow under vasodilator stress. The incremental prognostic value of TTE‐derived hCFV beyond conventional CFVR assessment in ANOCA patients remains uncertain. Accordingly, we designed this study to investigate whether hCFV obtained by TTE improves clinical risk stratification in ANOCA and provides additive prognostic information over established measures.

## 2 Results

### 2.1 Clinical Profiles and Flow Parameters in Patients With and Without MACE

During the follow‐up period (median 28.8 months [IQR 19.3–48.7]), a total of 33 MACE occurred in 27 patients. For time‐to‐event analyses, only the first event was counted; subsequent events were summarized descriptively. Among these patients, 12 (4.9%) had revascularization, 13 (5.3%) had myocardial infarction (MI), 6 (2.4%) were readmitted for heart failure (HF), and 2 (0.8%) were admitted for stroke. Patients were divided into two groups according to the follow‐up outcomes: MACEs and non‐MACEs. The MACE group was characterized by an older age, lower diastolic blood pressure, and a higher prevalence of statin and nitrate use compared to the non‐MACE group (*p* < 0.05). Notably, patients with MACE demonstrated lower hyperemic flow velocities (0.50 m/s [0.39, 0.58] vs. 0.65 m/s [0.53, 0.76], *p* < 0.001) and lower CFVR (2.24 [1.94, 3.06] vs. 2.93 [2.41, 3.36], *p* = 0.004), while resting flow velocities remained equivalent between the two groups (0.22 m/s [0.18, 0.27] vs. 0.21m/s [0.17, 0.26], *p* = 0.308). Besides, the MACE group exhibited markedly reduced LA‐Sr compared with the non‐MACE group (*p* < 0.05). No significant differences were observed in other baseline clinical variables and cardiovascular risk factors (Table 1).

**TABLE 1:** Comparative clinical characteristics and cardiac performances in ANOCA patients with and without MACE.

	Non‐MACE	MACE	*p*‐value
Clinical characteristics, *n* (%)	219/246 (89.1%)	27/246 (10.9%)	
Age (years)	57(51, 63)	61.5 (56, 66)	0.050
Female, *n* (%)	103 (46.8)	10 (38.5)	0.222
Comorbidities, *n* (%)			
Hypertension	107 (48.6)	10 (38.5)	0.326
Hyperlipidemia	117 (53.2)	17 (65.4)	0.237
Diabetes	48 (21.8)	5 (19.2)	0.762
Obesity	21 (9.5)	3 (11.5)	0.746
Smoking	78 (35.5)	9 (34.6)	0.933
Vital signs and physical characteristics			
Body mass index (kg/m^2^)	25 (23.3, 27.3)	24 (23.2, 27.7)	0.497
Body surface area (m^2^)	1.8 (1.6, 1.9)	1.7 (1.6, 1.8)	0.589
Heart Rate (bpm)	70 (62, 77)	71.5 (68, 78)	0.316
Systolic blood pressure (mmHg)	132.9 ± 15.3	128.3 ± 11.3	0.137
Diastolic blood pressure (mmHg)	80 (75, 86.5)	77 (73, 79)	0.022
Rate‐pressure product	9352 (7844, 10,295)	9039 (8352, 9709)	0.819
Coronary blood flow velocity (m/s)			
Rest flow velocity	0.22 (0.18, 0.27)	0.21 (0.17, 0.26)	0.308
Hyperemic flow velocity	0.65 (0.53, 0.76)	0.50 (0.39, 0.58)	< 0.001
CFVR	2.9 (2.4, 3.4)	2.2 (1.9, 3.1)	0.004
Medications, *n* (%)			
Aspirin	110 (50)	16 (61.5)	0.266
β‐Blocker	91 (41.4)	12 (46.2)	0.640
Calcium‐channel blocker	51 (23.2)	7 (26.9)	0.671
Statin	120 (54.5)	22 (84.6)	0.003
ACE‐inhibitor or ARB	87 (39.5)	6 (23.1)	0.306
Nitrates	53 (24.1)	12 (46.2)	0.016
Coronary evaluation modality (*n*, %)			
Coronary angiography	143 (65.3)	14 (51.9)	0.081
Coronary CT angiography	76 (34.7)	13 (48.1)	
Cardiac performance, *n* (%)	169/191 (88.5%)	22/191 (11.5%)	
Cardiac structure			
LV end‐diastolic dimension (mm)	45 (43, 49)	45 (42, 48)	0.413
Interventricular septal thickness (mm)	11 (9, 12)	10 (9, 11)	0.382
LV posterior wall thickness (mm)	10 (8, 11)	9 (8, 10)	0.594
LV mass index (g/m^2^)	93.6 (79.2, 108.4)	85.8 (76.5, 98.5)	0.141
LV end‐diastolic volume index (mL/m^2^)	41.1 (36.1, 47.9)	41.7 (31.1, 45.7)	0.277
Left atrium volume index (mL/m^2^)	24 (18.6, 31)	23.7 (19.4, 29)	0.605
Systolic/diastolic function			
LVEF (%)	64.9 ± 6.5	65.7 ± 7.8	0.580
LVGLS (%)	−21.4 ± 3.6	−20.9 ± 3.8	0.581
Peak strain dispersion (ms)	41 (30, 52)	46 (37, 58)	0.123
LA‐Sr (%)	36 (31, 43)	32 (26, 38)	0.027
LA‐Scd (%)	−17 (−22, −12)	−15.5 (−18, −10)	0.106
LA‐Sct (%)	−18 (−22, −15)	−17 (−20, −14)	0.307
Left atrial ejection fraction (%)	60.7 ± 10.7	58.5 ± 13.1	0.369
Left atrial function index	59.3 (40.8, 77.9)	54.9 (44.9, 77.2)	0.905
Average‐e’ (cm/s)	8 (6.5, 9)	7.8 (6, 8.5)	0.398
E/e’	8.4 (7.1, 9.9)	8.9 (7.3, 10.5)	0.460

*Note*: Data are presented as mean ± SD, medians [Q1, Q3], and number (percentage).

Abbreviations: CFVR, coronary flow velocity reserve; E, early diastolic transmittal flow velocity; e’, early diastolic mitral annular velocity; LA‐Scd, left atrial conduit strain; LA‐Sct, left atrial contraction strain; LA‐Sr, left atrial reservoir strain; LVEF, left ventricular ejection fraction; LVGLS, left ventricular global longitudinal strain; MACE, major adverse cardiovascular events.

### 2.2 Clinical Features and Prognostic Outcomes Stratified by CFVR

The clinical features of 246 patients with ANOCA, stratified by CFVR, are outlined in Table S1. Among the patients, 35.8% (88/246) demonstrated reduced CFVR (CFVR < 2.5), while 64.2% (158/246) exhibited normal CFVR (CFVR ≥ 2.5). Notably, patients with reduced CFVR had significantly higher heart rate, resting flow velocity, E/e’ values, and aspirin usage in comparison to those with normal CFVR. Conversely, hyperemic flow velocity, LA‐Sr, LA‐Scd, LVGLS, and average e’ were significantly lower in patients with reduced CFVR (Table S1).

Consistent with previous findings, patients with lower CFVR exhibited poorer prognosis. Kaplan–Meier survival curves further illustrated a markedly increased risk of MACE among patients with reduced CFVR compared to those with normal CFVR (Figure S1). Cox multivariate analysis identified reduced CFVR as a strong predictor of MACE risk in the ANOCA population (adjusted HR = 3.16, *p* = 0.005).

### 2.3 Definition of the hCFV Threshold

Given the differences in hyperemic flow velocities between the preserved‐ and reduced‐CFVR subgroups, we explored potential hCFV thresholds. Discrimination was modest in preserved CFVR (Figure S2A); therefore, threshold definition and subsequent risk stratification focused on the reduced‐CFVR subgroup.

The ROC analysis indicated that in patients with reduced CFVR, an hCFV ≤ 0.44 m/s provided useful risk stratification for MACE, with a sensitivity of 64.71% and a specificity of 71.83%. Specifically, the MACE rates were 35.5% for patients with hCFV ≤ 0.44 m/s compared to 10.5% for those with hCFV > 0.44 m/s (Figure S2). Furthermore, multivariate Cox analysis revealed that reduced hCFV was significantly associated with an increased probability of MACE in reduced CFVR patients (adjusted HR = 6.56, *p* < 0.001). The greater MACE rate in such patients was primarily caused by the high risk of admission for HF and MI (Figure S3).

### 2.4 Phenotypic Stratification by CFVR and hCFV: Distinct Flow Profiles With Similar Risk Factors and Comparable LV Dysfunction

To further characterize patient heterogeneity, patients were categorized into three groups according to CFVR and hCFV. Group A, which consisted of 64.2% of the cohort, exhibited a normal CFVR of ≥ 2.5. In contrast, Group B, which accounted for 23.2% of the patients, had a CFVR < 2.5 but maintained a high hCFV of > 0.44 m/s. Group C represented 12.6% of the cohort and was characterized by both low CFVR (< 2.5) and low hCFV (≤ 0.44 m/s). Although the groups differed in their CFVR and hCFV values, the distribution of cardiovascular risk factors was similar across all three groups. Both Group B and Group C exhibited reduced CFVR, but the underlying causes were different. In Group B, the hyperemic coronary blood flow velocity was maintained, but the resting coronary flow velocity was significantly increased. In contrast, in Group C, the coronary blood flow velocity was significantly reduced both at rest and under maximal hyperemia (Table 2).

**TABLE 2:** Baseline characteristics and physiological differences in patients with ANOCA, according to CFVR and hCFV.

	Group A (CFVR ≥ 2.5)	Group B (CFVR < 2.5, hCFV > 0.44 m/s)	Group C (CFVR < 2.5, hCFV ≤ 0.44 m/s)	*p*‐value
Clinical characteristics, *n* (%)	158 (64.2)	57 (23.2)	31 (12.6)	
Age (years)	56.1 ± 9.4	58.1 ± 9.9	58.1 ± 9.9	0.293
Female, *n* (%)	68 (43)	32 (56.1)	13 (41.9)	0.210
Comorbidities, *n* (%)				
Hypertension	75 (47.5)	27 (47.4)	15 (48.4)	0.995
Hyperlipidemia	79 (50)	35 (61.4)	20 (64.5)	0.162
Diabetes	32 (20.3)	13 (22.8)	8 (25.8)	0.762
Obesity	18 (11.4)	4 (7)	2 (6.5)	0.509
Smoking	59 (37.3)	16 (28.1)	12 (38.7)	0.417
Vital signs and physical characteristics				
Body mass index (kg/m^2^)	25.4 ± 3.2	24.8 ± 3.8	25.8 ± 3.2	0.389
Body surface area (m^2^)	1.8 ± 0.2	1.7 ± 0.2	1.8 ± 0.2	0.089
Heart rate (bpm)	69.2 ± 9.9	74.8 ± 15^†^	70.4 ± 11.1	0.009
Systolic blood pressure (mmHg)	132.4 ± 15.1	133.1 ± 16.1	131.7 ± 12.1	0.909
Diastolic blood pressure (mmHg)	80.5 ± 10.5	80.5 ± 9.3	78.2 ± 6.7	0.473
Rate‐pressure product	9066 (7839, 10,033)	9605 (8379, 10,710)	9100 (8447, 10,027)	0.081
Medications, *n* (%)				
Aspirin	73 (46.2)	31 (54.4)	22 (71) ^†^	0.036
β‐Blocker	63 (39.9)	27 (47.4)	13 (41.9)	0.617
Calcium‐channel blocker	36 (22.8)	13 (22.8)	9 (29)	0.746
Statin	88 (55.7)	31 (54.4)	23 (74.2)	0.137
ACE‐inhibitor or ARB	56 (35.4)	26 (45.6)	11 (35.5)	0.382
Nitrates	40 (25.3)	17 (29.8)	8 (25.8)	0.801
Coronary evaluation modality (*n*, %)				
Coronary angiography	97 (61.4)	42 (73.7)	17 (54.8)	0.146
Coronary CT angiography	61 (38.6)	15 (26.3)	14 (45.2)	
Coronary blood flow velocity				
Resting flow velocity (m/s)	0.2 (0.17, 0.24)	0.31 (0.26, 0.35) ^†^	0.2 (0.17, 0.22) ^‡^	< 0.001
Hyperemic flow velocity (m/s)	0.68 (0.57, 0.77)	0.63 (0.56, 0.78)	0.4 (0.37, 0.43) ^†‡^	< 0.001
CFVR	3.2 (2.9, 3.7)	2.2 (2.1, 2.4) ^†^	2 (1.8, 2.2) ^†^	< 0.001
Cardiac function, *n* (%)	122/191 (63.9)	43/191 (22.5)	26/191 (13.6)	
Cardiac structure				
LV end‐diastolic dimension (mm)	45.5 (42, 49)	45 (42, 50)	45 (44, 47)	0.908
Interventricular septal thickness (mm)	11 (9, 12)	11 (10, 12)	10 (9, 11)	0.132
LV posterior wall thickness (mm)	10 (8, 11)	10 (9, 11)	9 (8, 10)	0.211
LV end‐diastolic volume index (mL/m^2^)	40.6 ± 9.6	44.8 ± 12.4	38.9 ± 10.3	0.058
Left atrium volume index (mL/m^2^)	23.8 ± 8.4	23.8 ± 9.6	27.3 ± 12.2	0.117
Systolic/diastolic function				
LVEF (%)	65.3 ± 6.2	65 ± 6.5	64.1 ± 6.8	0.156
LVGLS (%)	−22 ± 3.7	−20.6 ± 3.2^†^	−19.6 ± 2.9^†^	0.001
Peak strain dispersion (ms)	42.1 ± 18.4	45 ± 15.7	44 ± 18.3	0.617
LA‐Sr (%)	38.5 ± 10.3	35.2 ± 9.1	28.3 ± 11.7^†‡^	< 0.001
LA‐Scd (%)	−18.8 ± 7.7	−16.3 ± 6.5	−13.1 ± 7.8^†^	0.002
LA‐Sct (%)	−19.6 ± 6.7	−19 ± 5.8	−15.3 ± 6.3^†‡^	0.011
Left atrial ejection fraction (%)	61.5 ± 10.9	60.3 ± 10.3	56.4 ± 12.9	0.117
Left atrial function index	60.9 (43.8, 77.9)	63.5 (43.1, 82.2)	48 (37.7, 83.7)	0.405
Average‐e’ (cm/s)	8 (7, 9.3)	7.5 (6, 8) ^†^	7 (6, 8.5) ^†^	0.004
E/e’	8.2 (6.8, 9.4)	9.4 (8, 11.8) ^†^	8.7 (7.3, 10.7)	0.008

*Note*: Data are presented as mean ± SD, medians [Q1, Q3], and number (percentage).

Abbreviations: CFVR, coronary flow velocity reserve; E, early diastolic transmitral flow velocity; e’, early diastolic mitral annular velocity; LA‐Scd, left atrial conduit strain; LA‐Sct, left atrial contraction strain; LA‐Sr, left atrial reservoir strain; LV, left ventricular; LVEF, left ventricular ejection fraction; LVGLS, left ventricular global longitudinal strain.

^†^ *p* < 0.05 compared with group A; ^‡^ *p* < 0.05 compared with group B.

In terms of cardiac function, patients in both Groups B and C displayed a comparable reduction in LVGLS and average e’ compared to those in Group A. Additionally, patients in Group C exhibited significant decreases in left atrial strain parameters, including LA‐Sr and LA‐Sct, when compared to Group B (Table 2).

### 2.5 Risk Stratification With Graded MACE Across Phenotypes and Incremental Prognostic Value of hCFV

**FIGURE 1:**
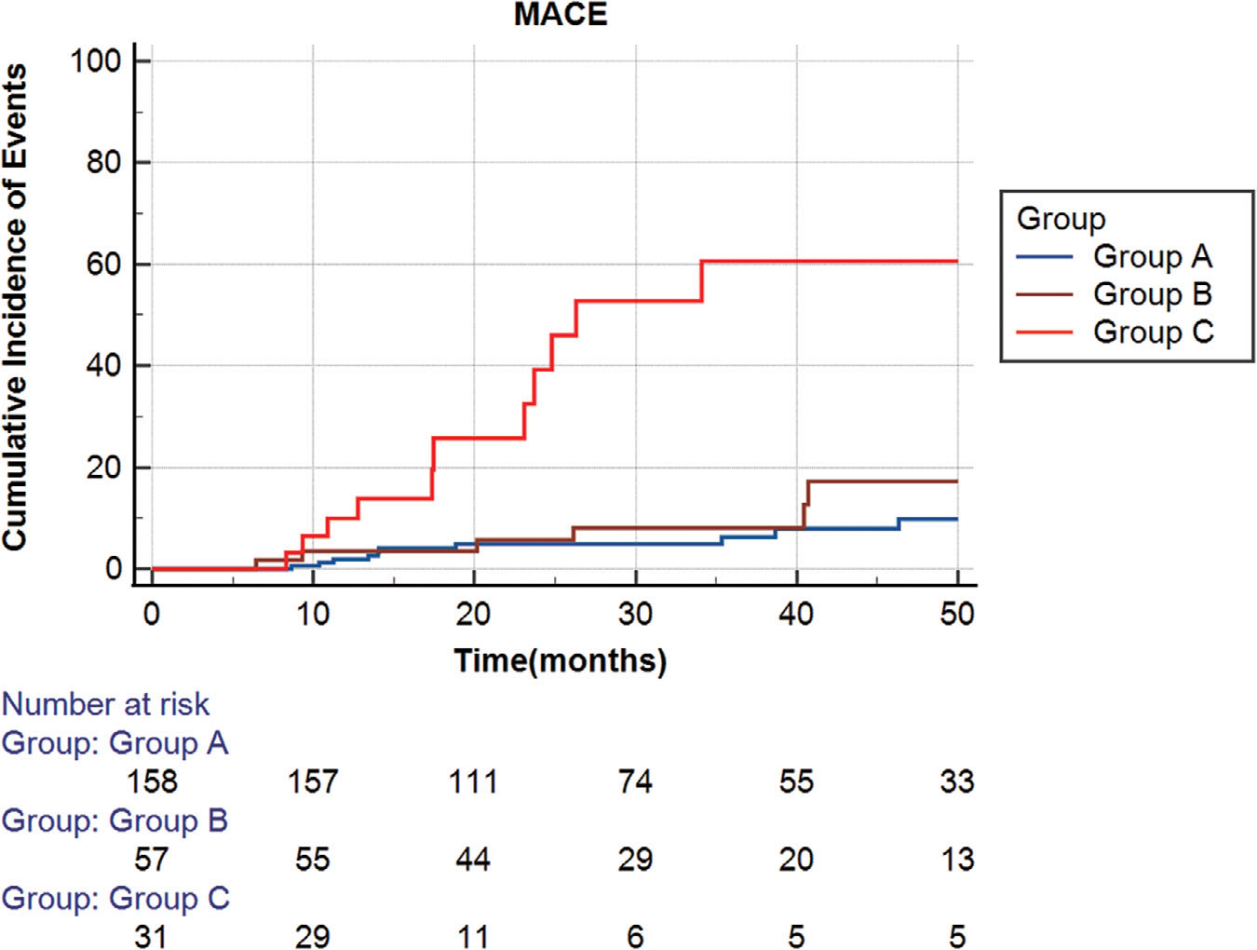
Kaplan–Meier curves depicting MACE events across three groups: Group A (CFVR ≥ 2.5), Group B (CFVR < 2.5, hCFV > 0.44 m/s), and Group C (CFVR < 2.5, hCFV ≤ 0.44 m/s). Median follow‐up was 28.8 months [IQR 19.3–48.7]. Abbreviations: CFVR, coronary flow velocity reserve; hCFV, hyperemic coronary flow velocity; MACE, major adverse cardiovascular events.

The incidence of MACE was 6.3%, 10.5%, and 35.5% in Groups A, B, and C, respectively (overall log‐rank *p* = 0.001) (Figure 1). When compared to Groups A and B, Group C had a considerably greater risk of MACE (vs. Group A: adjusted HR = 8.11, 95% CI: 3.316–19.833, *p* < 0.001; vs. Group B: HR = 6.567, 95% CI: 2.173–19.849, *p* = 0.001). The elevated rate of MACE in Group C was primarily caused by the increased risk of MI and admission for HF (Table 3).

**TABLE 3:** Clinical outcomes of patients with ANOCA according to CFVR and hCFV.

	Group A (CFVR ≥ 2.5)	Group B (CFVR < 2.5, hCFV > 0.44 m/s)	Group C (CFVR < 2.5, hCFV ≤ 0.44 m/s)	*p*‐value
Variable, *n* (%)	158 (64.2%)	57 (23.2%)	31 (12.6%)	
All‐cause death	0 (0)	0 (0)	0 (0)	NA
MI	3 (1.9)	4 (7)	6 (19.4)	0.001
Revascularization	4 (2.5)	5 (8.8)	3 (9.7)	0.070
Admission for HF	2 (1.3)	0 (0)	4 (12.9)	0.004
Admission for stroke	2 (1.3)	0 (0)	0 (0)	0.570
MACE	10 (6.3)	6 (10.5)	11 (35.5)	0.001

Abbreviations: HF, heart failure; MACE, major adverse cardiovascular events; MI, myocardial infarction.

**FIGURE 2:**
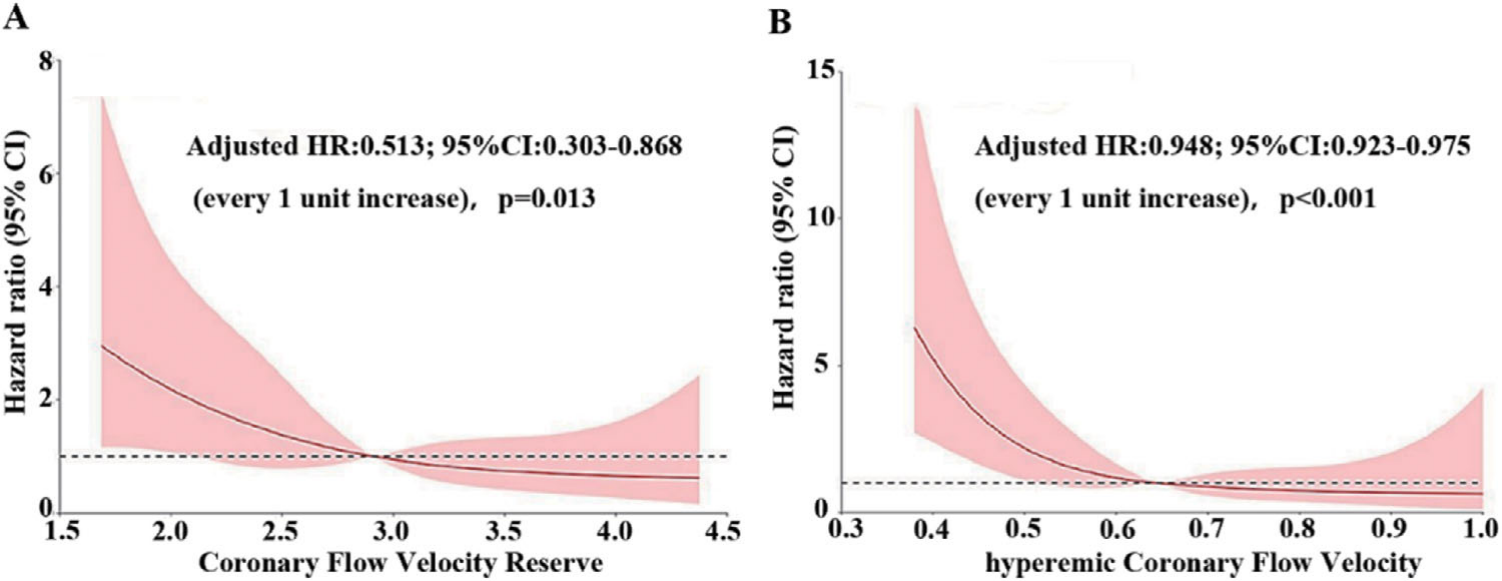
Association of CFVR and hCFV with risk of MACE. (A) Hazard ratio curve for CFVR; (B) hazard ratio curve for hCFV, adjusted for clinical covariates. Solid red lines represent HRs, and red shaded areas represent 95% CIs.

**FIGURE 3:**
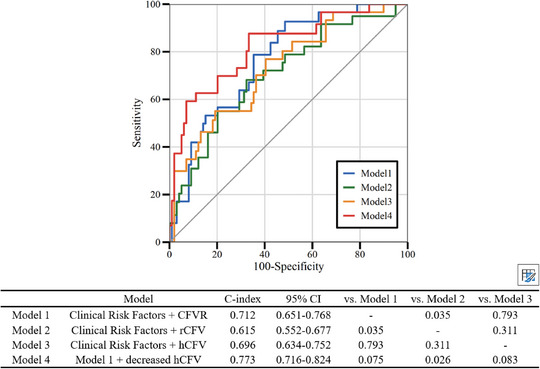
Time‐dependent ROC curves at 3 years were derived from Cox model linear predictors to evaluate discrimination accounting for censoring; the table summarizes Harrell's C‐index and pairwise comparisons. Model 1: Clinical risk Factors + CFVR; Model 2: Clinical risk Factors + resting coronary flow velocity; Model 3: Clinical risk Factors + hCFV; Model 4: Model 1+decreased hCFV (≤ 0.44 m/s) in patients with decreased CFVR (< 2.5). CFVR, coronary flow velocity reserve; hCFV: hyperemic coronary flow velocity; MACE, major adverse cardiovascular events.

In addition, as continuous variables, both CFVR and hCFV were significantly associated with the risk of MACE (Figure 2). An enhanced prognostic value was observed when the reduced hCFV was incorporated into the model that included clinical risk factors and CFVR for predicting adverse events, with a relative integrated discrimination improvement (IDI) of 0.05 (*p* = 0.011) and a category‐free net reclassification index (NRI) of 0.23 (*p* = 0.0023) (Figure 3), underscoring the incremental value of hCFV for risk stratification. Bootstrap internal validation (1000 resamples) demonstrated good calibration, with an optimism‐corrected calibration slope of 0.949.

### 2.6 Clinical Utility Assessment

**FIGURE 4:**
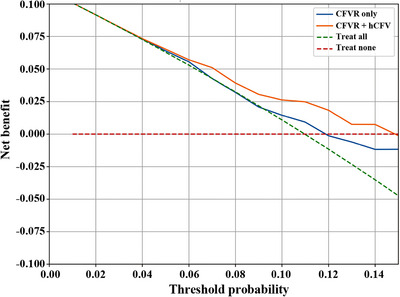
Decision curve analysis comparing CFVR only, CFVR + hCFV, treat‐all, and treat‐none strategies for predicting MACE at maximum follow‐up. The CFVR + hCFV model provides the greatest net benefit across threshold probabilities of 0–0.15. Abbreviations: CFVR, coronary flow velocity reserve; hCFV: hyperemic coronary flow velocity; MACE, major adverse cardiovascular events.

Decision curve analysis was performed to evaluate the clinical utility of adding hCFV to CFVR at maximum follow‐up (Figure 4). Given that long‐term event rates in ANOCA have been reported to be approximately 10%–15% and were comparable in our cohort, we evaluated clinically relevant threshold probabilities up to 0.15, corresponding to the range in which clinicians may reasonably consider further evaluation or intensified management [10]. Within this range, the CFVR plus hCFV model yielded greater net benefit than CFVR alone.

### 2.7 External Validation of CFVR‐ and hCFV‐Based Classification in ANOCA

The characteristics of the independent validation cohort are presented in Table S2. Patients in Group C showed the lowest LVGLS, the highest PSD, and the poorest left atrial function, with all comparisons being statistically significant (*p* < 0.05). In contrast, Group A exhibited the most favorable clinical and echocardiographic profiles. Like the derivation cohort, Group B also exhibited the highest resting coronary flow velocity in the validation cohort (*p* < 0.001; Table S2).

**FIGURE 5:**
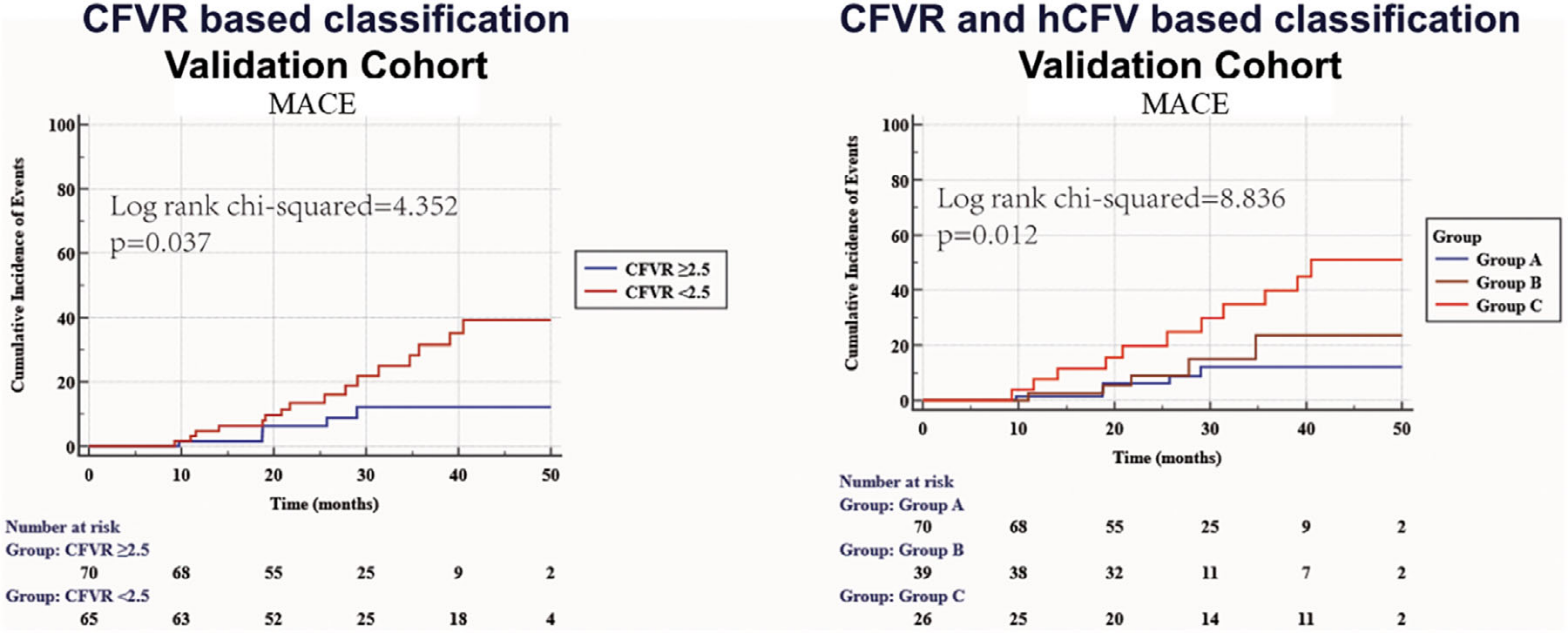
Kaplan–Meier curves for MACE in the validation cohort stratified by CFVR alone (A) and CFVR‐hCFV classification (B). Abbreviations: CFVR, coronary flow velocity reserve; hCFV: hyperemic coronary flow velocity; MACE, major adverse cardiovascular events.

During a median follow‐up period of 25.7 (21.1, 37.3) months, 16.3% of patients met the outcome. Group C had a considerably higher risk of MACE compared to Group A, with an adjusted HR of 3.79 (95% CI: 1.33–10.75, *p* = 0.001). Figure 5 illustrates the Kaplan–Meier curves for patients stratified by CFVR classification and by CFVR and hCFV classification. The CFVR‐ and hCFV‐based classification resulted in a wider separation of survival curves for MACE, as reflected by a larger log‐rank χ^2^ statistic (8.836 vs. 4.352). Additionally, an increased prognostic value was observed in the validation cohort. In the validation cohort, adding hCFV to CFVR and clinical covariates did not significantly improve the C‐index (*p* = 0.098). However, both the IDI (0.082, *p* = 0.018) and the NRI (0.474, *p* = 0.038) were statistically significant, indicating better risk stratification despite the nonsignificant change in overall concordance (Figure S4).

## 3 Discussion

In this study, we found that in patients with ANOCA, integrating hCFV with CFVR identified distinct phenotypes with graded risk of MACE. Both CFVR and hCFV were associated with prognostic outcomes when analyzed as continuous measures, and the combined CFVR‐hCFV classification stratified risk more sharply than CFVR alone. Similar results were also observed in an independent validation cohort. To our knowledge, this is the first study to evaluate the prognostic value of echocardiography‐derived hCFV for further risk stratification in patients with ANOCA.

### 3.1 Current Methods and Issues in Diagnosing ANOCA

Previous studies have established that patients with ANOCA are at a higher risk of cardiovascular events compared to asymptomatic healthy populations which increased attention to ANOCA [11]. While CFVR‐guided risk stratification has demonstrated effectiveness [3, 12], its reliance on resting flow velocity and hemodynamics may be a weakness of this parameter. For instance, Johnson et al. suggested that in scenarios involving anxiety or elevated myocardial workload, baseline flow may be elevated even when maximal flow is normal. Consequently, CFVR may appear low despite the absence of ischemic signs or symptoms. In our research, the elevated resting flow velocity observed in patients from Group B is partly attributable to their higher heart rates, a phenomenon also noted in other studies. Lee et al. reported that, among patients with ANOCA, resting heart rate was the sole independent predictor of resting mean transit time [13]. Furthermore, the relationship between symptoms and reduced CFVR remains unsubstantiated and weak. In the iPOWER (Improving Diagnosis and Treatment of Women with Angina Pectoris and Microvascular Disease) study, which focused on female patients with ANOCA, Jakob et al. found that a reduced CFVR was linked to a higher incidence of adverse cardiovascular events but did not result in more severe angina symptoms [14].

To more clearly differentiate these patients, the Coronary Vasomotor Disorders International Study Group proposed diagnostic criteria for impaired coronary microvascular function, which include depressed CFVR and elevated coronary microvascular resistance (defined as index of microcirculatory resistance ≥ 25 or hyperemic microcirculatory resistance ≥ 1.9) [15, 16]. Although this represents a significant advancement in establishing a research framework for ANOCA, its implementation in routine clinical practice remains limited for several reasons. First, the measurement of microvascular resistance is invasive, costly, time consuming, and technically complex that necessitates cooperation between the patient and operator. Second, recent studies have questioned the relevance of microvascular resistance. For example, Hong et al. reported that CFVR was the primary prognostic factor in symptomatic patients with intermediate but functionally nonsignificant epicardial stenosis, while the index of microcirculatory resistance showed no significant association with the risk of the primary outcome (cardiovascular death or admission for HF) after adjusting for CFVR [5]. In addition, in the ILIAS (Inclusive Invasive Physiological Assessment in Angina Syndromes) study, Boerhut et al. reported that hyperemic microcirculatory resistance did not correlate with 5‐year MACE. Among patients with reduced CFVR, MACE risk did not differ between those with elevated versus normal hyperemic microvascular resistance [6].

Therefore, there remains a need for a more practical and prognostically informative noninvasive stratification approach in ANOCA. The combination of CFVR with hyperemic flow, which encompasses all relevant flow characteristics of the vasculature under investigation, was first proposed by Johnson and Gould [17, 18], and this method has been validated in catheterization laboratories. In their study, Van de Hoef et al. reported that combining invasively measured hyperemic peak blood flow velocity with CFVR improved risk stratification for patients at risk of major adverse clinical events compared to CFVR alone, whereas other ischemic tests, such as fractional flow reserve and hyperemic microcirculatory resistance, did not yield such incremental values [19]. These findings prompted us to investigate the prognostic significance of TTE‐derived coronary flow velocity and to establish the optimal cutoff value for clinical application.

### 3.2 Different Physiologic Profiles Based on CFVR and hCFV

The combined assessment of CFVR and hCFV identified two distinct physiologic patterns among patients with reduced CFVR. Group B showed reduced CFVR despite preserved hCFV, suggesting that the lower CFVR in these patients may be related to higher resting flow rather than an impaired hyperemic flow capacity. Previous studies have reported increased nitric oxide synthase activity in similar patients, which may reflect a partially vasodilated state at rest [20].

In contrast, Group C showed both reduced CFVR and low hCFV, a pattern more consistent with impaired microvascular vasodilatory capacity. This group also had a substantially higher event rate and less favorable atrial and ventricular strain profiles in our study. Taken together, these findings suggest that hCFV may provide additional prognostic information beyond CFVR alone, particularly in patients with reduced CFVR arising from different underlying physiologic mechanisms [21, 22].

### 3.3 Prognostic Impact of Different Phenotypic Patterns of ANOCA According to CFVR and hCFV

CFVR is undoubtedly an important prognostic indicator for ANOCA patients, but a decrease in CFVR may also be due to diffuse, balanced epicardial coronary atherosclerosis or myocardial diseases [23]. In a study of 139 patients with ANOCA, Lee et al. reported that endothelial dysfunction was present in 44% of patients, and microvascular impairment occurred in 21% [13]. Additionally, intravascular imaging revealed diffuse nonobstructive atherosclerosis in all these patients [24], suggesting a need to reconsider contemporary microvascular‐centered diagnostic strategies for this complex disease and to pursue comprehensive re‐stratification for patients with ANOCA. For example, in a study based on PET‐myocardial blood flow, Ziadi et al. found that combining myocardial blood flow can significantly enhance the prognostic value of myocardial flow reserve [25].

In the current study, patients exhibited different coronary flow patterns based on CFVR and hCFV. A reduced CFVR was associated with a 3.1‐fold greater risk of MACE compared to normal CFVR, aligning with findings from previous studies [26]. In patients exhibiting low CFVR, the risk of primary outcomes was 6.6 times greater in those with reduced hCFV compared to those without, and these patients also demonstrated worse cardiac function. Furthermore, a reduced hCFV was significantly associated with an increased risk of admission for HF and MI, though the incidence of ischemia‐driven revascularization was comparable. This suggests that the heightened myocardial injuries in Group C may be attributed to diffuse atherosclerosis or microvascular disease rather than to focal obstruction. Prior studies have confirmed that patients with low CFVR and reduced hCFV tend to exhibit elevated levels of N‐terminal pro‐brain natriuretic peptide and experience more frequent exercise‐induced ischemia [20], potentially contributing to adverse outcomes.

Adding hCFV to CFVR produced significant improvements in reclassification (IDI and NRI), whereas the change in C‐index was not statistically significant in the validation cohort. This divergence is expected: C‐index averages concordance over all pairs and is relatively insensitive to re‐ranking within clinically relevant probability ranges. In contrast, IDI/NRI quantify how much individual risk estimates move in the right direction; their significance indicates meaningful patient‐level risk redistribution despite small changes in global concordance. Clinically, this means incorporating hCFV helps identify higher risk patients who would otherwise be misclassified by CFVR plus clinical factors alone.

### 3.4 Clinical Implications

Our findings suggest that combined assessment of CFVR and hCFV may help refine risk stratification in patients with ANOCA. Patients with reduced CFVR but preserved hCFV and those with both reduced CFVR and low hCFV showed different flow patterns and different risks of adverse events. This classification may therefore provide additional information beyond CFVR alone in routine noninvasive evaluation. However, the present study was observational, and the clinical utility of this classification should be further evaluated in prospective studies.

### 3.5 Limitations

This study has several limitations. First, while the analysis included a well‐characterized population and an independent validation cohort with a high follow‐up rate, the overall sample size was moderate. Besides, we only used the distal LAD coronary artery for the assessment of CFVR and hCFV, potential heterogeneity in the right or circumflex arteries was not captured. Prior study from Cortigiani et al. suggests that LAD‐derived CFVR best identifies genuinely high‐risk patients, whereas right coronary CFVR is less informative [27].

Nevertheless, multivessel assessment could provide added granularity and warrants future study.

## 4 Conclusion

This study suggests that TTE‐derived hCFV provides incremental prognostic information beyond CFVR in patients with ANOCA. Combined assessment of CFVR and hCFV may improve noninvasive risk stratification in this population.

## 5 Materials and Methods

### 5.1 Study Population

We enrolled 280 consecutive patients with ANOCA at Qilu Hospital, Shandong University (May 2018–October 2022). Inclusion required (a) angina symptoms; (b) evidence of ischemia (ECG changes, abnormal exercise ECG, or perfusion defects on SPECT); and (c) coronary angiography (CAG) or coronary CT angiography (CCTA) showing < 50% stenosis. Coronary anatomy was assessed by either CAG or CCTA. Transthoracic Doppler testing was performed within 30 days after CAG/CCTA confirmed nonobstructive coronary arteries (< 50% stenosis). All participants underwent evaluation at their initial presentation of angina with objective evidence of ischemia, and none had a prior history of MI, diagnosed coronary artery disease, or coronary revascularization. All participants underwent adenosine‐stress transthoracic Doppler echocardiography to measure CFVR. Exclusion criteria were adenosine intolerance, inadequate image quality, chronic kidney disease (eGFR < 30 mL/min/1.73 m^2^), valvular disease >mild, cardiomyopathy, LVEF < 50%, and atrial fibrillation or other severe arrhythmias. Thirty‐four patients were excluded (renal insufficiency *n* = 2; poor image quality *n* = 9; malignancy or rheumatic disease *n* = 12; lost to follow‐up *n* = 11), leaving 246 for analysis. Caffeine and medications that could affect coronary hemodynamics were withheld for 24 h (e.g., nitrates, β‐blockers, calcium‐channel blockers, and other antianginal agents), when clinically feasible. The study was approved by the institutional ethics committee (KYLL202008019); all patients provided informed consent.

### 5.2 Transthoracic Echocardiographic Examination

Comprehensive two‐dimensional echocardiography was performed using a GE Vivid E95/E9 system with an M5S probe (1.5–4.6 MHz). Parasternal long‐axis and apical four‐, three‐, and two‐chamber views were acquired in the left lateral decubitus position for ≥ 3 cardiac cycles at 50–80 frames/s. Standard parameters included LV end‐diastolic dimension, interventricular septal and posterior‐wall thicknesses, LV mass index, LV end‐diastolic volume index (LVEDVi), left atrial volume index (LAVI), and LVEF by the biplane Simpson method. Left atrial ejection fraction (LAEF) was calculated as (LAVmax – LAVmin)/LAVmax, and the LA functional index (LAFI) as (LAEF × LVOT − VTI)/LAVI [28]. Transmitral E velocity was recorded by pulsed‐wave Doppler; early diastolic mitral annular velocities (e′) were obtained at septal and lateral annuli by tissue Doppler and averaged.

Speckle‐tracking analysis using the Automated Function Imaging (AFI) module in EchoPAC (v204) yielded LV global longitudinal strain (LVGLS), time‐to‐peak strain dispersion (PSD), and left‐atrial reservoir (LA‐Sr), conduit (LA‐Scd), and contraction (LA‐Sct) strains [29, 30]. Endocardial borders were traced and regions of interest auto‐tracked across standardized segments (17‐segment LV model). All strain curves were reviewed by two experienced echocardiographers.

### 5.3 Coronary Flow Velocity Reserve and Flow Velocity Acquisition

All patients enrolled in the study underwent adenosine‐based assessment of CFVR. The protocol employed a previously published and validated method for quantifying CFVR during the adenosine stress test [31]. For this study, we utilized the GE Vivid E9 and E95 ultrasound systems equipped with an M5S transducer. The sample volume was set to 2 mm, and the low velocity reject setting was adjusted to 3.8 cm/s. Adenosine was administered intravenously at a dose of 0.14 mg/kg/min for 6 min, with continuous monitoring of the electrocardiogram throughout the procedure. The mid‐distal segment of the left anterior descending (LAD) branch was identified in the interventricular sulcus, and its flow spectrum was recorded using color Doppler echocardiography continuously. CFVR was defined as the ratio of the maximum diastolic flow velocity during the hyperemic state to the maximum diastolic flow velocity in the basal state. In the present study, reduced CFVR was defined a priori as CFVR < 2.5, consistent with previous research [12, 32]. hCFV was defined as the maximum diastolic flow velocity during the hyperemic state.

### 5.4 Follow‐up and Clinical Assessment

Clinical data for this study were collected from outpatient clinic visits and medical records. Each patient was followed up for a median 28.8 months (IQR 19.3–48.7) by their physician at Qilu Hospital of Shandong University through telephone contact or outpatient visits. The primary endpoint of our study was MACE, which encompassed a range of events, including all‐cause death, nonfatal MI, ischemia‐driven revascularization, hospital admission for HF, and stroke. All‐cause death refers to any death, regardless of the underlying cause. Nonfatal MI was characterized by an increase in the creatine kinase‐myocardial band or troponin level accompanied by symptoms of myocardial ischemia. Ischemia‐driven revascularization referred to percutaneous coronary intervention (PCI) or coronary artery bypass grafting performed in response to MI or recurrent angina pectoris. Hospitalization for HF was defined as an inpatient admission due to signs and symptoms of heart failure with elevated N‐terminal pro‐brain natriuretic peptide and necessitated diuretics or inotrope therapy [33]. Hospital admission for stroke was contingent upon the presence of objective evidence indicating either intracranial hemorrhage or ischemic stroke [11].

### 5.5 External Validation Cohort

We enrolled additional 135 clinically ANOCA patients followed at Qilu Hospital of Shandong University, China. The validation cohort was independently assembled, and there was no patient overlap between the derivation and validation datasets. Inclusion and exclusion criteria used for the exploratory cohort were also applied for the validation group. Echocardiographic examinations were acquired using the same standardized protocol and performed by an investigator blinded to the exploratory group analyses.

### 5.6 Statistical Analysis

Categorical variables are presented as counts (percentages) and compared using the chi‐square test or Fisher's exact test, as appropriate. The distribution of continuous variables was assessed with the Kolmogorov–Smirnov test. Normally distributed variables are expressed as mean ± SD and compared by *t*‐tests or analysis of variance; non‐normal variables are expressed as median [Q1, Q3] and compared by the Kruskal–Wallis test.

To determine the hCFV threshold most predictive of MACE, receiver‐operating characteristic (ROC) analysis was performed in MedCalc; the area under the curve (AUC) was estimated (DeLong method), and the cut‐off was chosen by maximizing the Youden index. This ROC‐derived threshold was used to classify patients into low versus high hCFV groups (we also analyzed hCFV continuously). CFVR was analyzed both continuously and categorically.

Time‐to‐event analyses used Cox proportional hazards models from the index echocardiography to first MACE or last contact. Clinically prespecified covariates were age, sex, heart rate, hypertension, diabetes, current smoking, and obesity, in addition to the exposures (CFVR and hCFV). The proportional hazards assumption was assessed using Schoenfeld residuals, and no major violation was observed. Internal validation was performed using bootstrap resampling (1000 resamples) to assess model calibration and quantify optimism; the optimism‐corrected calibration slope was reported. Discrimination was assessed using Harrell's C‐index. Incremental prognostic value of adding hCFV (and the CFVR–hCFV combination) to clinical and echocardiographic variables was evaluated by IDI and category‐free NRI. Kaplan–Meier curves were compared by the log‐rank test. Time‐to‐event analyses were based on the first event per patient; recurrent events were reported descriptively. Two‐sided *p* < 0.05 was considered statistically significant. Analyses were conducted in SPSS version 26 and R 4.3.1; figures were produced in MedCalc 19.04.

## Author Contributions

Quande Liu and Guihua Jiang contributed to the conception and design of the study, methodology, formal analysis, data interpretation, and writing – original draft. Mingjun Xu, Jichen Pan, Chenghu Guo, Yichun Zhou, Meng Zhang, and Yu Zhang contributed to data acquisition and data analysis. Yun Zhang, Mengmeng Li, and Mei Zhang contributed to critical revision of the manuscript for important intellectual content and final approval. All authors have read and approved the final manuscript.

## Funding

This work was supported by the National Key Research and Development Program of China (2022YFC3602403), Shandong Provincial Key Research and Development Program (2020ZLYS05), Fundamental Research Funds for the Central Universities (2023QNTD003), and Shandong Provincial Natural Science Foundation (ZR2023MH058).

## Ethics Statement

The study was approved by the Ethics Committee of Scientific Research of Shandong University Qilu Hospital (KYLL202008019) and conducted as per the Declaration of Helsinki. Informed consent was obtained from all participants in the study.

## Conflicts of Interest

All authors declare no conflicts of interest.

## Supporting information




**Supporting File 1**: mco270731‐sup‐0001‐SuppMat.Docx

## Data Availability

Summary data are provided in the article and Supporting Information. De‐identified participant‐level data are available from the corresponding author upon reasonable request with appropriate institutional approvals due to ethical restrictions.
