# Supplementary material for: The Incremental Prognostic Value of Hyperemic Coronary Flow Velocity in Patients with Angina and Nonobstructive Coronary Artery Disease
Source: MedComm (2020). 2026 Apr 12;7(4):e70731. doi: 10.1002/mco2.70731 (PMC13070199; doi:10.1002/mco2.70731)
Supplement: Supplementary file 1 — Supporting File 1: mco270731‐sup‐0001‐SuppMat.Docx [file MCO2-7-e70731-s001.docx]

**Supplementary Information**

**The Incremental** **Prognostic Value of Hyperemic Coronary Flow Velocity in Patients with** **Angina and Nonobstructive Coronary Artery Disease**

Quande Liu^1^, Guihua Jiang^1^, Mingjun Xu^1^, Jichen Pan^1^, Chenghu Guo^1^, Yichun Zhou^1^, Meng Zhang^1^, Yu Zhang^1^, Yun Zhang^1^, Mengmeng Li^1^*, Mei Zhang^1^*

**^1^** National Key Laboratory for Innovation and Transformation of Luobing Theory; The Key Laboratory of Cardiovascular Remodeling and Function Research, Chinese Ministry of Education, Chinese National Health Commission and Chinese Academy of Medical Sciences; Department of Cardiology, Qilu Hospital of Shandong University, Jinan, China

**Co-first authors:**

Quande Liu, [lqd16688206038@163.com](mailto:lqd16688206038@163.com)

Guihua Jiang, jgh13953117218@163.com

*** Corresponding authors:**

Mei Zhang, [daixh@vip.sina.com](mailto:daixh@vip.sina.com)

Mengmeng Li, [zpyzlmm90128@163.com](mailto:zpyzlmm90128@163.com)

**Table S1** Baseline Characteristics and Physiological Differences in Patients With ANOCA, According to CFVR

**Table S2** Baseline Characteristics and Physiological Differences in External Validation cohort, according to CFVR and hCFV

**Figure S1** Cumulative incidence of the major adverse cardiovascular events, according to CFVR

**Figure S2** hCFV cutoff for ANOCA patients by exploratory ROC analysis

**Figure S3** Kaplan-Meier survival curves of MACE in low CFVR patients according to hCFV

**Figure S~~4~~** Incremental prognostic impact of hCFV to predict MACE in Validation Cohort

**Supplementary Tables**

**Table S1 Baseline Characteristics and Physiological Differences in Patients With ANOCA, According to CFVR**

|  | **Total** | **Normal CFVR (CFVR ≥ 2.5)** | **Decreased CFVR**  **(CFVR < 2.5)** | ***p*-value** |
| --- | --- | --- | --- | --- |
| **Clinical characteristics, n (%)** | **246** | **158/246 (64.2%)** | **88/246 (35.8%)** |  |
| Age (years) | 57.5 (51, 64) | 57(50, 62) | 59 (52, 65) | 0.080 |
| Female, n (%) | 113 (45.9) | 68 (43.4) | 45 (51.1) | 0.222 |
| Comorbidities, n (%) |  |  |  |  |
| Hypertension | 115 (46.7) | 75 (47.5) | 40 (45.5) | 0.762 |
| Hyperlipidemia | 134 (51.5) | 79 (50) | 55 (62.5) | 0.059 |
| Diabetes | 53 (21.5) | 32 (20.3) | 21 (23.9) | 0.509 |
| Obesity (BMI>30 kg/m^2^) | 24(9.8) | 18 (11.4) | 6 (6.8) | 0.246 |
| Smoking | 87 (35.4) | 59 (37.3) | 28 (31.8) | 0.385 |
| Vital signs and physical characteristics |  |  |  |  |
| Body mass index (kg/m^2^) | 24.9 (23.2, 27.4) | 25 (23.3, 27.3) | 24.7 (23, 27.4) | 0.494 |
| Body surface area (m^2^) | 1.8 (1.6, 1.9) | 1.8 (1.7, 1.9) | 1.7 (1.6, 1.9) | 0.079 |
| Heart Rate (bpm) | 70.5 (62, 77) | 68.5 (62, 74) | 72.5 (64, 81) | 0.011 |
| Systolic blood pressure (mmHg) | 132.4±15 | 132.4±15.1 | 132.6±14.7 | 0.697 |
| Diastolic blood pressure (mmHg) | 80 (75, 85) | 80 (74, 87) | 79 (75, 84) | 0.509 |
| Rate-pressure product | 9314 (7980, 10218) | 9066 (7839, 10033) | 9372 (8389, 10501) | 0.052 |
| Medications, n (%) |  |  |  |  |
| Aspirin | 126 (51.2) | 73 (46.2) | 53 (60.2) | 0.046 |
| β-blocker | 103 (41.9) | 63 (39.9) | 40 (45.5) | 0.395 |
| Calcium-channel blocker | 58 (23.6) | 36 (22.8) | 22 (25) | 0.695 |
| Statin | 142 (57.7) | 88 (55.7) | 54 (61.4) | 0.388 |
| ACE-inhibitor or ARB | 93 (37.8) | 56 (35.4) | 37 (42) | 0.306 |
| Nitrates | 65 (26.4) | 40 (25.3) | 25 (28.4) | 0.598 |
| Coronary evaluation modality (n, %) |  |  |  |  |
| Coronary angiography | 156 (63.4) | 97 (61.4) | 59 (67) | 0.378 |
| Coronary CT angiography | 90 (36.6) | 61 (38.6) | 29 (32.9) |  |
| Coronary blood flow velocity (m/s) |  |  |  |  |
| Resting flow velocity | 0.22 (0.18, 0.27) | 0.20 (0.17, 0.24) | 0.26 (0.21, 0.33) | <0.001 |
| Hyperemic flow velocity | 0.64 (0.5, 0.75) | 0.68 (0.57, 0.77) | 0.56 (0.43, 0.68) | <0.001 |
| CFVR | 2.9 (2.3, 3.3) | 3.2 (2.9, 3.7) | 2.2 (1.9, 2.4) | <0.001 |
| **Cardiac function, n (%)** | **191** | **122/191 (63.9%)** | **69/191 (36.1%)** |  |
| Cardiac structure |  |  |  |  |
| LV end-diastolic dimension (mm) | 45 (42.5, 49) | 45.5 (42, 49) | 45 (43, 48) | 0.733 |
| Interventricular septal thickness (mm) | 11 (9, 12) | 11 (9, 12) | 10 (9, 12) | 0.533 |
| LV posterior wall thickness (mm) | 10 (8, 11) | 10 (8, 11) | 10 (8, 10) | 0.455 |
| LV mass index (g/m^2^) | 91.5 (78.9, 107.8) | 89.7 (76.6, 106.7) | 96.5 (81.9, 114.5) | 0.190 |
| LV end-diastolic volume index (ml/m^2^) | 41.2 (35.5, 47.4) | 40.8 (36, 47) | 41.9 (34.6, 47.5) | 0.818 |
| Left atrium volume index (ml/m^2^) | 24 (18.7, 30.6) | 23.9 (18.4, 30) | 24.1 (19.5, 33.9) | 0.249 |
| Systolic/Diastolic function |  |  |  |  |
| LVEF (%) | 65±6.5 | 65.3±6.2 | 64.5±7 | 0.482 |
| LVGLS (%) | -21.3±3.6 | -22±3.7 | -20.1±3.2 | <0.001 |
| Peak strain dispersion (ms) | 41 (30, 53) | 40 (30, 52) | 46 (32, 57) | 0.159 |
| LA-Sr (%) | 36 (31, 42) | 37 (32, 44) | 32 (26, 39) | <0.001 |
| LA-Scd (%) | -17 (-22, -12) | -17.7 (-23, -13) | -15 (-18, -10) | 0.001 |
| LA-Sct (%) | -18 (-22, -15) | -18 (-22, -15) | -17 (-20, -14) | 0.068 |
| Left atrial ejection fraction (%) | 60.4±11 | 61.5±10.9 | 58.6±10.9 | 0.079 |
| Left atrial function index | 58.3 (40.8, 77.9) | 60.9 (41.5, 77.9) | 55.2 (38.6, 77.6) | 0.286 |
| Average-e’ (cm/s) | 8 (6.5, 9) | 8 (7, 9.5) | 7.5 (6, 8.5) | <0.001 |
| E/e’ | 8.5 (7.1, 10) | 8.2 (6.8, 9.4) | 8.9 (7.8, 11.3) | 0.001 |

Data are presented as mean±SD, medians [Q1, Q3], and number (percentage).

Abbreviations: CFVR, coronary flow velocity reserve; E, early diastolic transmitral flow velocity; e’, early diastolic mitral annular velocity; LA-Scd: left atrial conduit strain; LA-Sct, left atrial contraction strain; LA-Sr: left atrial reservoir strain; LV, left ventricular; LVEF, left ventricular ejection fraction; LVGLS, left ventricular global longitudinal strain.

**Table S2 Baseline Characteristics and Physiological Differences in External Validation cohort, according to CFVR and hCFV**

|  | **Group A**  **(CFVR ≥ 2.5)** | **Group B**  **(CFVR < 2.5, hCFV > 0.44 m/s)** | **Group C**  **(CFVR < 2.5, hCFV ≤ 0.44 m/s)** | ***p*-value** |  |
| --- | --- | --- | --- | --- | --- |
| **Clinical characteristics, n (%)** | **70 (51.8)** | **39 (28.9)** | **26 (19.3)** |  |  |
| Age (years) | 56.7±8.6 | 59.1±7.9^†^ | 60.5±8.2^†^ | 0.003 |  |
| Female, n (%) | 31 (44.3) | 16 (41) | 11 (42.3) | 0.944 |  |
| Comorbidities, n (%) |  |  |  |  |  |
| Hypertension | 26 (37.1) | 19 (48.7) ^†^ | 7 (27) ^†‡^ | 0.019 |  |
| Hyperlipidemia | 28 (40) | 13 (33.3) | 13 (50) | 0.459 |  |
| Diabetes | 12 (17.1) | 10 (25.6) | 10 (38.5) ^†^ | 0.032 |  |
| Obesity (BMI>30 kg/m^2^) | 6 (8.5) | 5 (12.8) | 3 (11.5) | 0.102 |  |
| Smoking | 26 (37.1) | 12 (30.8) | 13 (50) | 0.315 |  |
| Vital signs and physical characteristics |  |  |  |  |  |
| Heart Rate (bpm) | 70 (65, 77) | 78 (62, 87.5) ^†^ | 72 (57, 78) ^‡^ | 0.021 |  |
| Systolic blood pressure (mmHg) | 132.4±15.6 | 136±18.9 | 133.7±8 | 0.518 |  |
| Diastolic blood pressure (mmHg) | 81.6±10 | 80.2±7.7 | 79.2±6.3 | 0.453 |  |
| Rate-pressure product | 9366 (8378, 10230) | 10318 (8288, 13112) | 9144 (7980, 10218) | 0.075 |  |
| Medications, n (%) | |  |  |  |  |
| Aspirin | | 26 (37.1) | 19 (48.7) ^†^ | 15 (57.7) ^†^ | 0.002 |
| β-blocker | | 16 (22.8) | 19 (48.7) ^†^ | 11 (42.3) ^†^ | 0.001 |
| Calcium-channel blocker | | 11 (15.7) | 7 (17.9) | 8 (30.7) | 0.071 |
| Statin | | 34 (48.6) | 20 (51.3) ^†^ | 18 (69.2) ^†^ | 0.001 |
| ACE-inhibitor or ARB | | 20 (28.6) | 13 (33.3) | 7 (26.9) | 0.103 |
| Nitrates | | 15 (21.4) | 8 (20.5) | 6 (23.1) | 0.369 |
| Coronary blood flow velocity (m/s) |  |  |  |  |  |
| Rest flow velocity | 0.22±0.06 | 0.29±0.09 ^†^ | 0.18±0.04 ^‡^ | <0.001 |  |
| Peak flow velocity | 0.69±0.17 | 0.62±0.14 ^†^ | 0.38±0.06 ^†‡^ | <0.001 |  |
| CFVR | 3.1 (2.9, 3.4) | 2.3 (2, 2.4) ^†^ | 2.2 (2, 2.4) ^†^ | <0.001 |  |
| Cardiac structure |  |  |  |  |  |
| LV end-diastolic dimension(mm) | 46.5±5.8 | 46.4±5.9 | 47.5±7.1 | 0.760 |  |
| Interventricular septal thickness (mm) | 10.5±2.4 | 10.7±1.7 | 10.2±2.9 | 0.146 |  |
| LV posterior wall thickness (mm) | 8.5 (8, 10) | 9.5 (7, 10) | 8 (7, 11) | 0.283 |  |
| LV mass index (g/m^2^) | 89±19.4 | 92.6±26.8 | 91.6±35.9 | 0.654 |  |
| LV end-diastolic volume index (ml/m^2^) | 40.5 (37.4, 46.6) | 40 (36.8, 42.6) | 41.3 (31.1, 46.4) | 0.491 |  |
| Left atrium volume index (ml/m^2^) | 24 (18.5, 29.9) | 20.4 (11.9, 36.3) | 28.8 (20.2, 35.4) | 0.320 |  |
| Systolic/Diastolic function |  |  |  |  |  |
| LVEF (%) | 65.2±6.8 | 63±8 | 64.7±5.3 | 0.337 |  |
| LVGLS (%) | -22.1±3.5 | -19.8±3.7 | -16.8±2.8 ^†^ | 0.007 |  |
| Peak strain dispersion (ms) | 37.9±15.1 | 44.2±17.4 | 46.9±16.9 ^†^ | 0.046 |  |
| LA-Sr (%) | 37.3±7.8 | 34.5±7.5 | 29.2±11.8 ^†‡^ | 0.003 |  |
| LA-Scd (%) | -17.7±6.2 | -15.7±5.5 | -14.1±7.9 | 0.193 |  |
| LA-Sct (%) | -19.5±6.5 | -19.9±6.2 | -15.2±8.1 ^†‡^ | 0.003 |  |
| Left atrial ejection fraction (%) | 61.7±11.5 | 58.7±11.7 | 55.5±15.9 | 0.092 |  |
| Left atrial function index | 59.5 (40.1, 77.9) | 56.4 (36.6, 84.2) | 47.5 (33.9, 55.6) ^†‡^ | 0.032 |  |
| Average-e’ (cm/s) | 8±2 | 7±2 ^†^ | 7±1 ^†^ | <0.001 |  |
| E/e’ | 8.1 (6.8, 9.3) | 8.7 (8.3, 11) | 9.4 (8, 11.8) | 0.210 |  |

^†^p<0.05 compared with group A; ^‡^p<0.05 compared with group B

Data are presented as mean±SD, medians [Q1, Q3], and number (percentage).

Abbreviations: CFVR, coronary flow velocity reserve; E, early diastolic transmitral flow velocity; e’, early diastolic mitral annular velocity; LA-Scd: left atrial conduit strain; LA-Sct, left atrial contraction strain; LA-Sr: left atrial reservoir strain; LV, left ventricular; LVEF, left ventricular ejection fraction; LVGLS, left ventricular global longitudinal strain.

**Supplementary Figures**

**
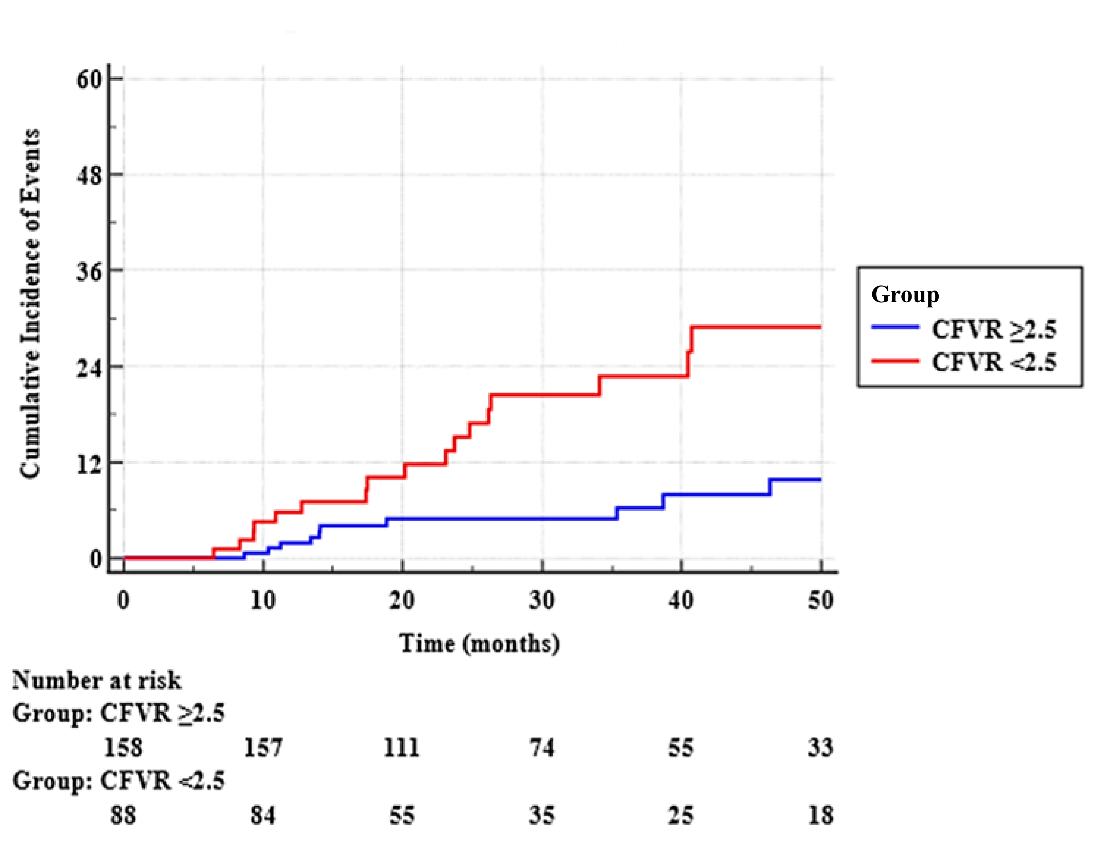
**

**Figure S1. Cumulative incidence of** **the major adverse cardiovascular events, according to CFVR.** Kaplan-Meier survival curves for patients stratified by CFVR (<2.5 vs. ≥2.5) during a median follow-up of 28.8 months.

Abbreviations: CFVR: coronary flow velocity reserve

**
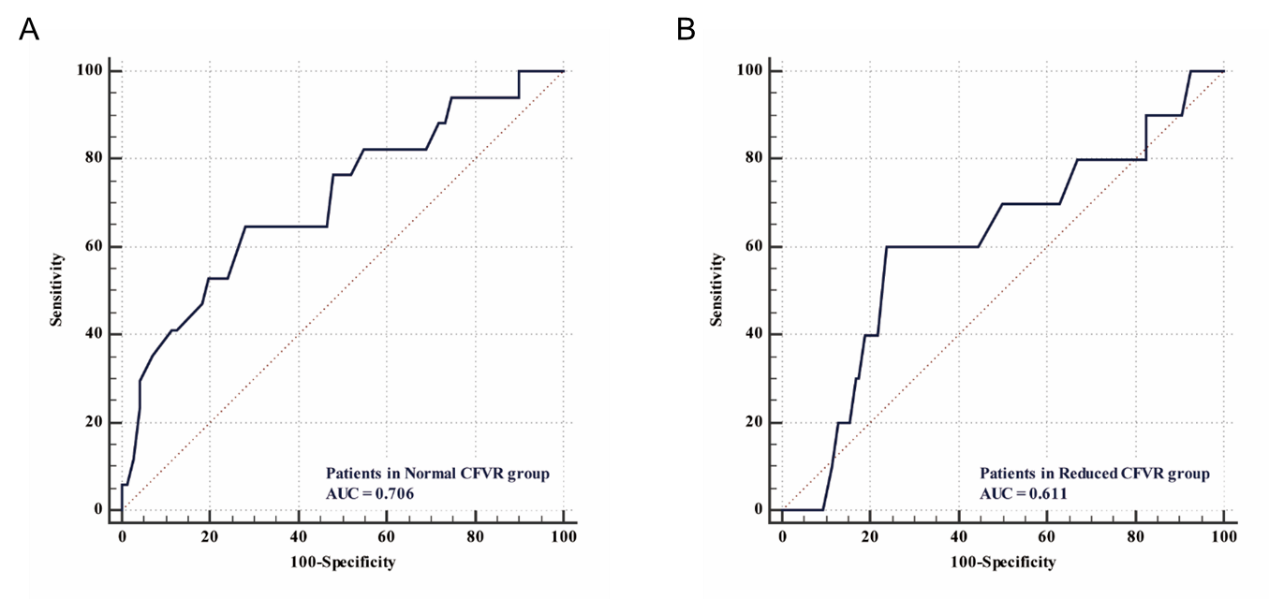
**

**Figure S2. hCFV cutoff for ANOCA patients by exploratory ROC analysis.** Receiver operating characteristic curves identifying the optimal hCFV thresholds for predicting MACE in (A) normal and (B) reduced CFVR groups.

Abbreviations: ANOCA, angina and nonobstructive coronary artery disease; CFVR, coronary flow velocity reserve; hCFV, hyperemic coronary flow velocity; MACE, major adverse cardiovascular events.

**
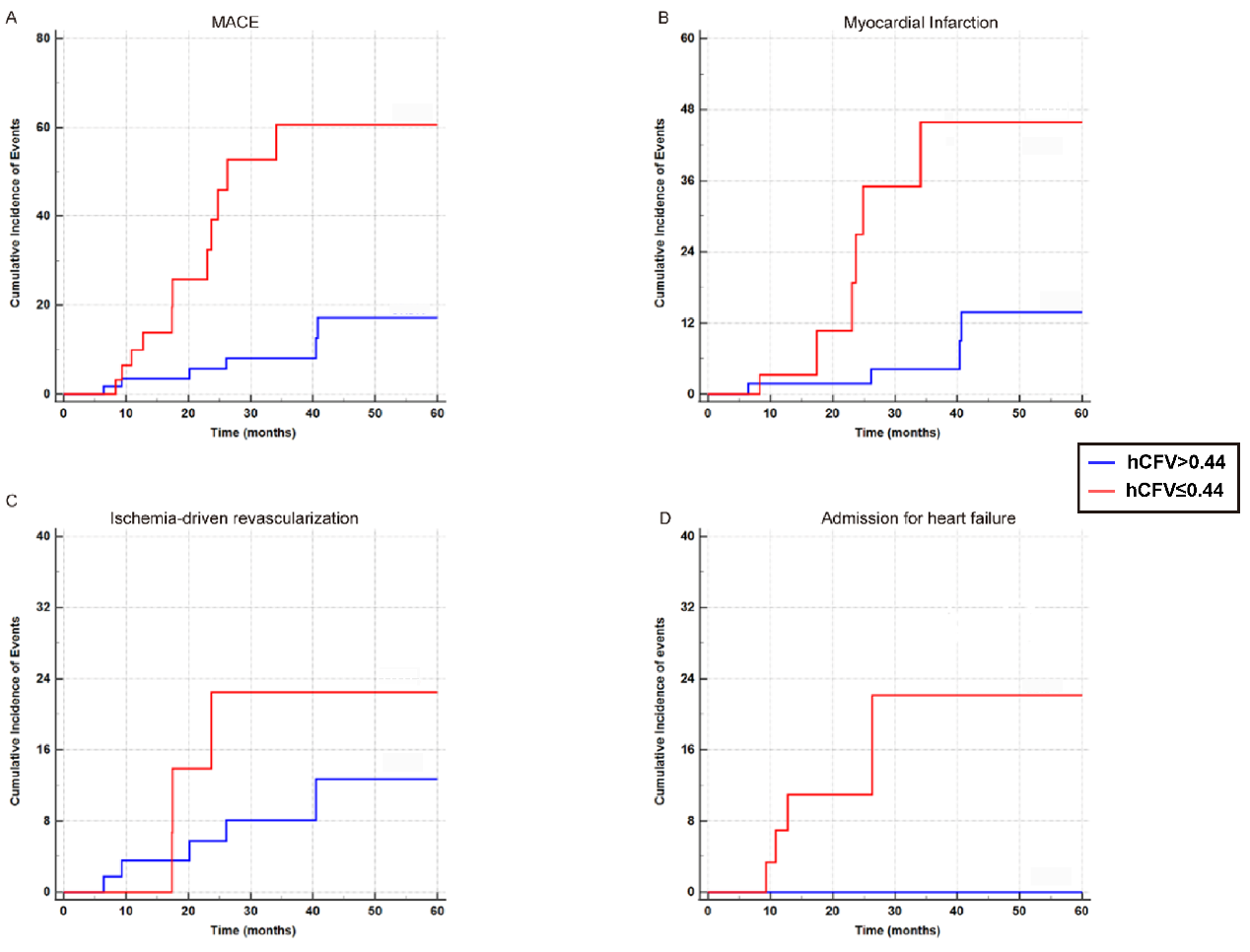
**

**Figure S3. Kaplan-Meier survival curves of MACE in low CFVR patients according to hCFV.** Kaplan-Meier curve is presented for the cumulative incidence of MACE (A), myocardial infarction (B), ischemia-driven revascularization (C), and admission for heart failure (D) according to hCFV. No deaths or hospital admissions for stroke in reduced CFVR patients.

Abbreviations: CFVR, coronary flow velocity reserve; hCFV, hyperemic coronary flow velocity; MACE, major adverse cardiovascular events.

**
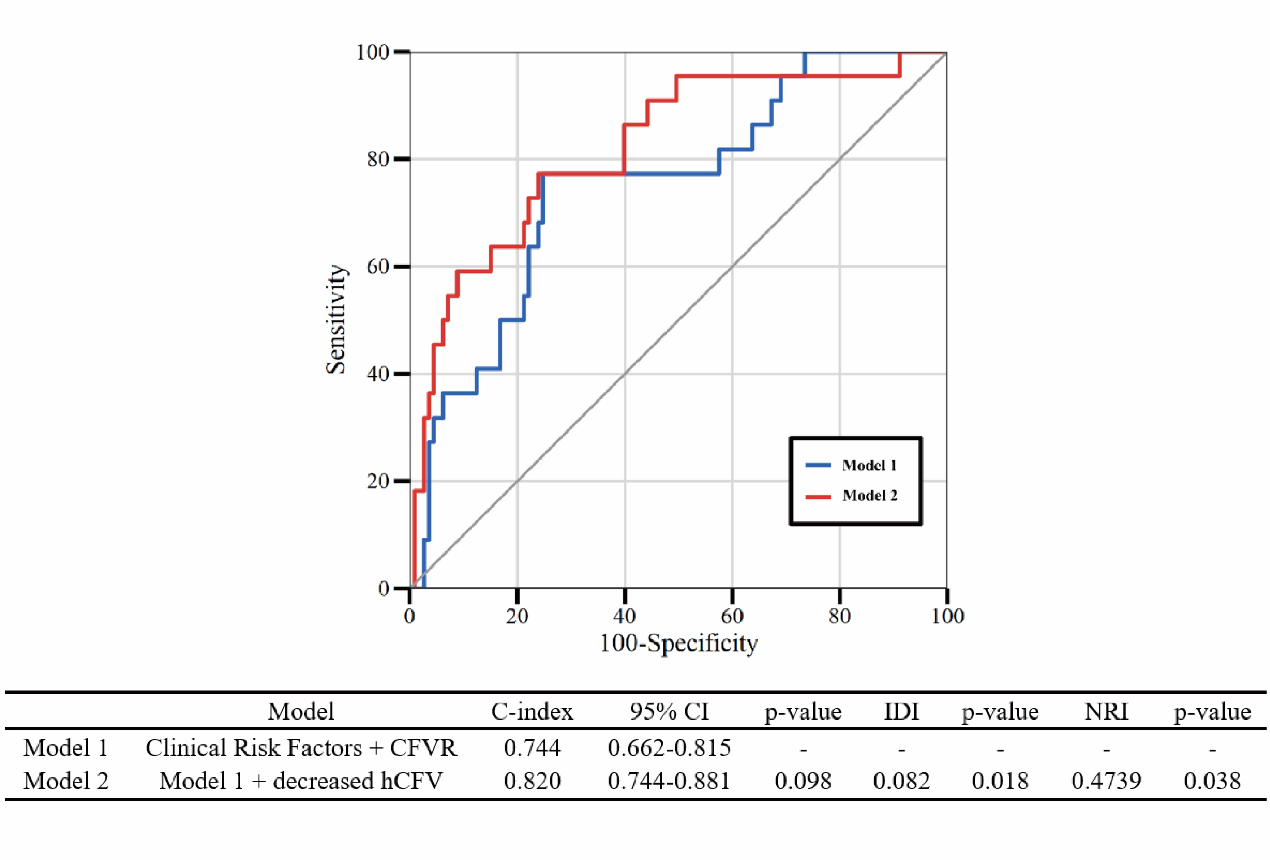
**

**Figure S~~4~~. Incremental prognostic impact of hCFV to predict MACE in Validation Cohort.** Abbreviations: CFVR, coronary flow velocity reserve; CI, confidence interval; hCFV, hyperemic coronary flow velocity; IDI, integrated discrimination improvement; NRI, net reclassification improvement.
